# Priming Effects in Boreal Black Spruce Forest Soils: Quantitative Evaluation and Sensitivity Analysis

**DOI:** 10.1371/journal.pone.0077880

**Published:** 2013-10-30

**Authors:** Zhaosheng Fan, Julie D. Jastrow, Chao Liang, Roser Matamala, Raymond Michael Miller

**Affiliations:** 1 Biosciences Division, Argonne National Laboratory, Argonne, Illinois, United States of America; DOE Pacific Northwest National Laboratory, United States of America

## Abstract

Laboratory studies show that introduction of fresh and easily decomposable organic carbon (OC) into soil-water systems can stimulate the decomposition of soil OC (SOC) via priming effects in temperate forests, shrublands, grasslands, and agro-ecosystems. However, priming effects are still not well understood in the field setting for temperate ecosystems and virtually nothing is known about priming effects (e.g., existence, frequency, and magnitude) in boreal ecosystems. In this study, a coupled dissolved OC (DOC) transport and microbial biomass dynamics model was developed to simultaneously simulate co-occurring hydrological, physical, and biological processes and their interactions in soil pore-water systems. The developed model was then used to examine the importance of priming effects in two black spruce forest soils, with and without underlying permafrost. Our simulations showed that priming effects were strongly controlled by the frequency and intensity of DOC input, with greater priming effects associated with greater DOC inputs. Sensitivity analyses indicated that priming effects were most sensitive to variations in the quality of SOC, followed by variations in microbial biomass dynamics (i.e., microbial death and maintenance respiration), highlighting the urgent need to better discern these key parameters in future experiments and to consider these dynamics in existing ecosystem models. Water movement carries DOC to deep soil layers that have high SOC stocks in boreal soils. Thus, greater priming effects were predicted for the site with favorable water movement than for the site with limited water flow, suggesting that priming effects might be accelerated for sites where permafrost degradation leads to the formation of dry thermokarst.

## Introduction

Priming effects are changes in the decomposition rate of soil organic carbon (SOC) [1] caused by (1) addition of easily decomposable substances (e.g., fresh and/or young SOC released from roots or litterfall) into the soil [2], [3], [4], [5] or (2) other interventions such as soil drying and rewetting processes [6], [7]. Priming effects can stimulate SOC decomposition [positive priming; 8,9,10,11] or suppress SOC decomposition [negative priming; 12,13,14] when fresh SOC and other nutrients are added to the soil environment. Possible mechanisms behind positive and negative priming effects have been well described and documented in Cheng et al. [15], Kuzyakov et al. [1], and Kuzyakov [16], [17].

Priming effects have been studied mostly in temperate forests, shrublands, grasslands, and agro-ecosystems. But studies on priming effects in the boreal and tundra ecosystems, which store approximately 35% of world’s terrestrial SOC [18], have been very limited in number. Laboratory incubation studies by Hartley et al. [19] showed that microbial respiration and SOC mineralization rates of sub-arctic mountain birch forest soils were stimulated after addition of glucose and/or glycine to the soil. Similarly, the growth-chamber studies of Loya et al. [20] suggested that leaching of carbon from leaves may have positive priming effects on the decomposition of leaf litter at the soil surface due to the stimulation of microbial activity. However, Loya et al. [20] also reported the presence of live roots may have negative priming effects on the decomposition of SOC and root litter due to a switch in the energy sources used for microbial activities from relatively old SOC to the more readily decomposable root-derived C.

Very few studies have evaluated the role of priming effects through modeling. Even for temperate ecosystems, most existing models on priming effects have been constructed at the laboratory scale and have focused on static environments (e.g., laboratory experiments). In addition, few process-based quantitative methods are currently available for evaluating priming effects under dynamic environments with water flow (i.e., field conditions) or for determining how climate and soil environmental factors control priming. However, dissolved organic carbon (DOC) plays a vitally important role in microbial biomass dynamics and subsequent priming effects by providing energy for microbial growth and maintenance [21], [22]. Thus, the production, fate, and transport of DOC in soil can have a great impact on microbial biomass dynamics, but to our best knowledge, these DOC dynamics have not been integrated into models of SOC priming effects.

In this study, a mechanistically sophisticated process-based model was developed to simulate coupled soil physical, hydrological, and biological processes and to examine the importance of priming effects in boreal forest soils. The model was developed with vertically resolved SOC layers to examine the processes that control the magnitude and dynamics of priming effects in one well-drained boreal forest soil without underlying permafrost and one moderately well-drained boreal forest soil with underlying permafrost. We believe that this model could be used as a tool to provide the insights needed to effectively design and plan future field experiments to investigate priming effects, especially in the high-latitude regions.

## Materials and Methods

### Site Descriptions

Two black spruce forest sites, one well-drained and one moderately well-drained, were used as prototypes to evaluate the potential importance of priming effects on SOC decomposition in boreal forest. Both sites were located within the Donnelly Flats region near Delta Junction, Alaska, USA, with mean annual precipitation of 305 mm and mean annual air temperature of -2.3°C [23]. The well-drained site (hereafter WD) was burned in 1921 and the moderately well-drained site (hereafter MWDp) was burned in 1880. For both sites, drainage condition was classified on the basis of soil characteristics (e.g., soil moisture condition, organic horizon thickness, and permafrost) and described in detail previously [24], [25], [26]. Briefly, WD has approximately 10 cm organic horizon thickness, unsaturated soil condition in the upper 75 cm, and no underlying permafrost [26]; while MWDp has approximately 20 cm organic horizon thickness, saturated soil condition at depths of 50–70 cm, and underlying permafrost [26].

**Figure 1:**
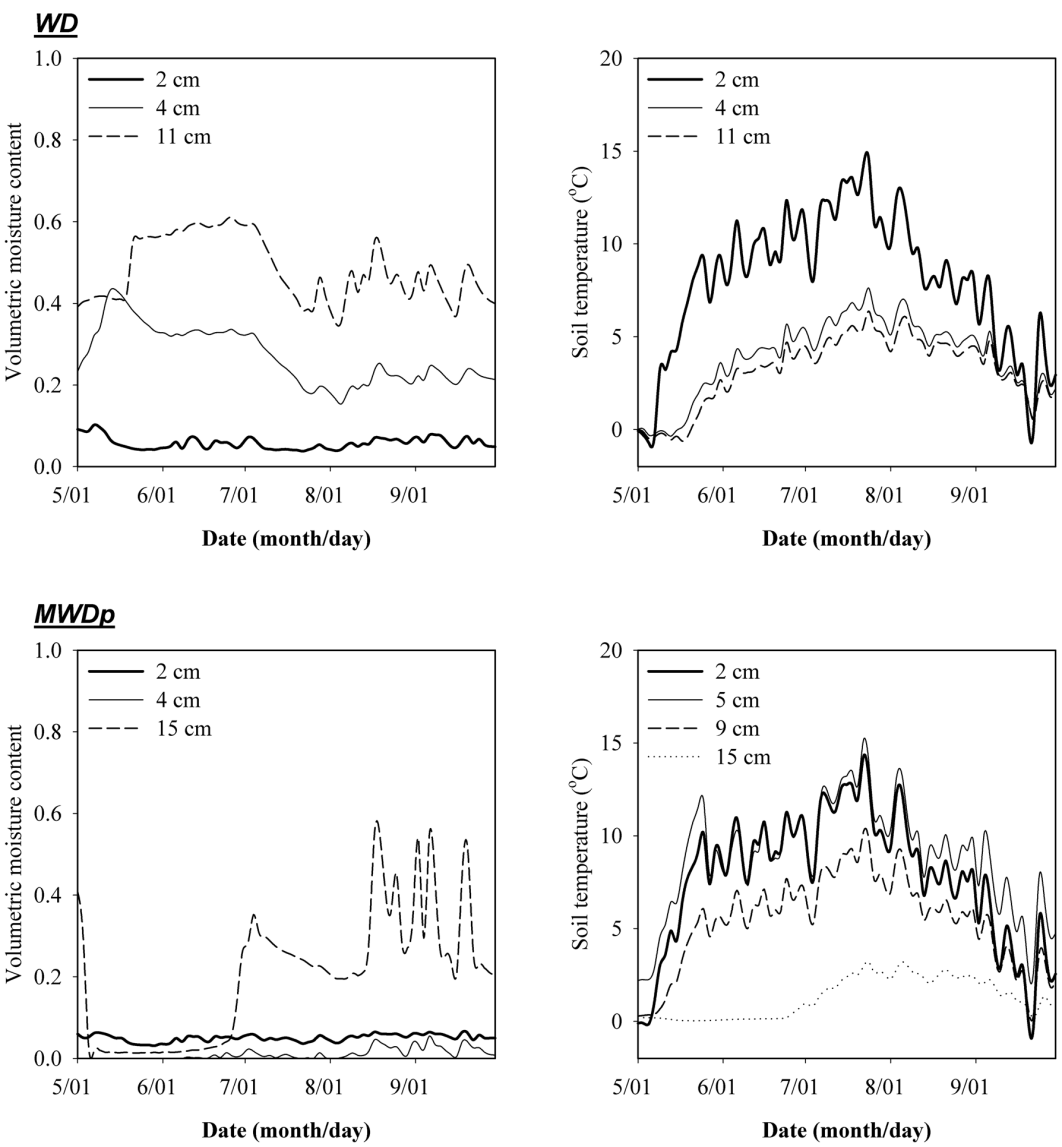
Soil moisture and temperature at measured soil depths in the well-drained site without permafrost (WD) and in the moderately well-drained site with permafrost (MWDp).

The vertical soil profile at the WD site contains live moss from 0–2 cm, dead moss and litter from 2 to 4 cm, slightly decomposed SOC and roots from 4 to 5 cm, moderately decomposed SOC from 5 to 11 cm, and mineral soil below 11 cm. The vertical soil profile at the MWDp site contains live moss from 0–1 cm, dead moss and litter from 1 to 3 cm, amorphous material from 3 to 14 cm, moderately decomposed SOC from 14 to 15 cm, well decomposed SOC with charcoal from 15 to 19 cm, and mineral soil below 19 cm. The mineral soil texture in both WD and MWDp is silt loam and the C:N ratio in the organic horizon decreased from 66 at soil surface to 29 at the base of organic horizon in WD and from 63 to 22 in MWDp [24]. The soil temperature and moisture in WD were measured hourly at 2, 4, 11, and 37 cm in 2002 (Figure 1). In MWDp, soil temperature was measured hourly at 2, 5, 9, and 11 cm and soil moisture at 2, 4, 15, and 25 cm in 2002 (Figure 1).

### Model Descriptions

**Figure 2:**
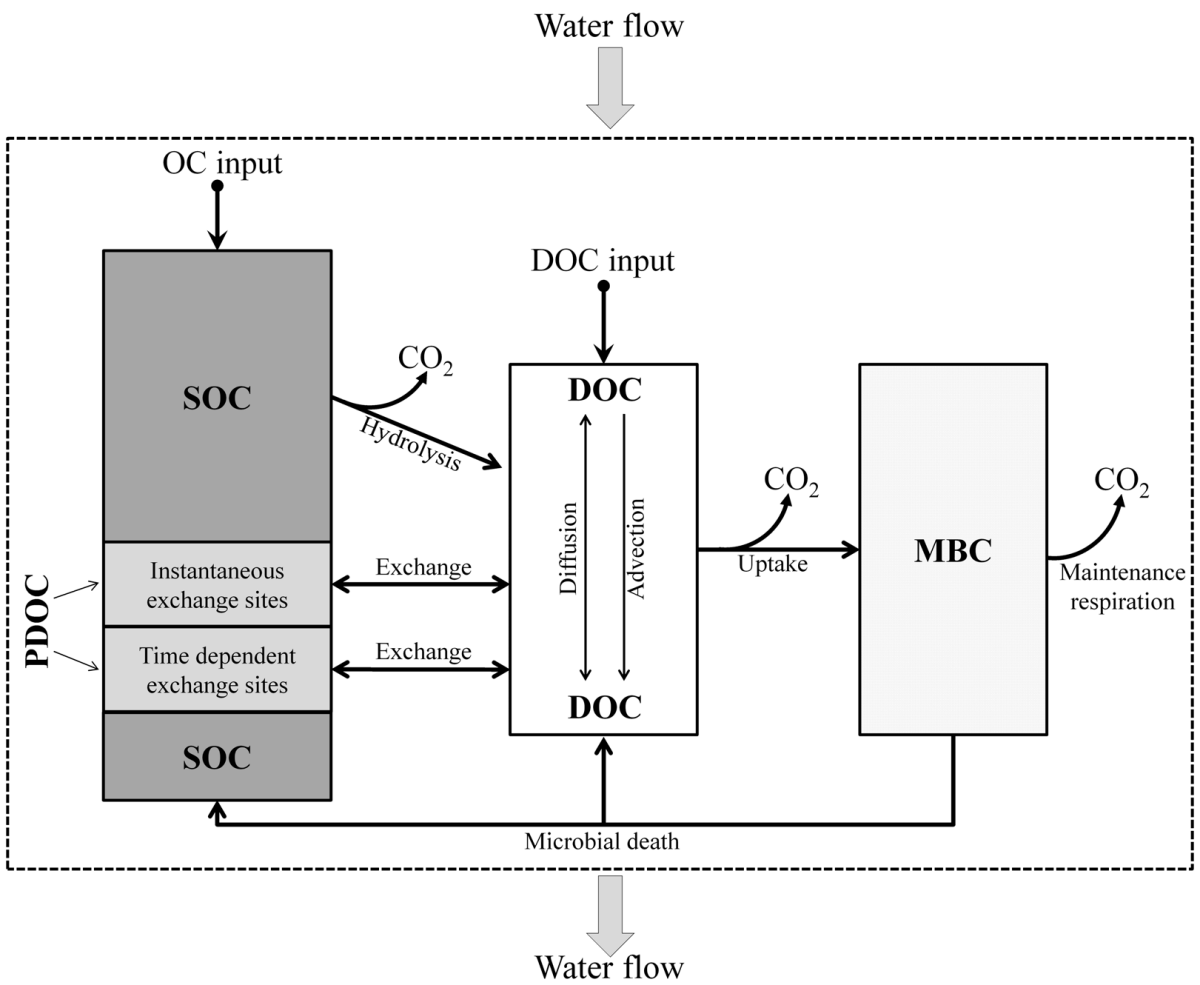
Schematic of the process-based model that was used to describe the simultaneously occurring production, fate, and transport of dissolved organic carbon and microbial biomass dynamics. DOC: dissolved organic matter; PDOC: potentially dissolvable OC that currently remains in the solid phase; SOC: soil organic matter; MBC: microbial biomass carbon.

To describe the coupled dynamics of SOC, DOC, and microbial biomass in the soil, a two-site chemical nonequilibrium fate and transport model with linear sorption/desorption was modified and used in this study [27]. The model concept is presented in Figure 2. The model consists of four OC pools: (1) SOC pool, (2) potentially dissolvable OC that currently remains in the solid phase (PDOC), (3) DOC, and (4) microbial biomass OC (MBC).

In our conceptual model, the MBC pool is separated from the SOC pool. It was assumed that MBC can be transferred to SOC and DOC pools upon microbial cell death (as microbial necromass). The decomposition of SOC, DOC, and MBC was simulated based on the studies of Blagodatsky et al. [28] where MBC was treated not only as an additional OC pool but also as a driving factor of OC decomposition. It is assumed that SOC is initially degraded by microbes through hydrolysis, which releases DOC. Once DOC is produced, it can be used as a substrate for microbial growth and respiration. A proportion of microbial necromass (i.e., dead microbial mass) is transferred to the soluble OC (i.e., DOC), while the remaining portion is transferred to the SOC pool. CO_2_ is released by hydrolysis of SOC to DOC, microbial uptake of DOC (i.e., microbial growth), and microbial maintenance respiration (Figure 2). Real priming effects (i.e., changes in decomposition of the SOC pool) are calculated as the extra CO_2_ released from the SOC pool through the hydrolysis process due to the introduction of DOC [28]. Because we are more interested in the role of real priming effects, apparent priming effects (i.e., extra CO_2_ released due to accelerated microbial metabolism with no accompanying change in SOC [5], [16], [29]) were not investigated here.

In the model, the soil column above the mineral soil is composed of three horizons: dead moss and slightly decomposed OC horizon, moderately decomposed OC horizon, and well decomposed OC horizon. Within each horizon, there are several layers of prescribed thickness (i.e., 1 cm) to numerically resolve the soil water and DOC dynamics. The underlying mineral soil was not considered in the model.

### DOC and PDOC Pools

The dynamics of the DOC and PDOC pools are quantitatively expressed by the following partial differential equation [27], [28], [30], [31]:
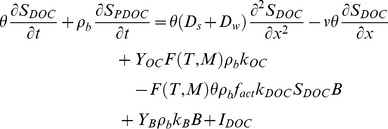
(1)where *θ* is the volumetric moisture content (cm^3^ cm^−3^), *S_DOC_* is the concentration of DOC in the aqueous phase (g DOC mL^−1^), *t* is the time (h), *ρ_b_* is the soil bulk density (g cm^−3^), *S_PDOC_* is the concentration of PDOC (g PDOC g^−1^ OC), *D_s_* is the hydrodynamic dispersion coefficient (cm^2^ h^−1^), *D_w_* is the diffusion coefficient of DOC in water (cm^2^ h^−1^), *x* is the soil depth (cm), *v* is the pore water velocity (cm h^−1^), *Y_OC_* is the fraction of hydrolyzed SOC that transfers to the DOC pool during the decomposition of SOC (unitless), *F*(*T, M*) is the scaling factor for calculating the impacts of soil temperature (*T*) and moisture (*M*) on the decomposition rate of DOC, *k_OC_* is the hydrolysis rate coefficient of SOC (h^−1^), *B* is the MBC (g OC), *k_DOC_* is the decomposition rate of DOC (h^−1^), *f_act_* is the microbial activity function ranging from 0 to 1 (unitless), *Y_B_* is the fraction of microbial necromass that transfers to the DOC pool during turnover (unitless), *k_B_* is the microbial death rate (h^−1^), and *I_DOC_* is the external DOC input rate (g h^−1^). The first and second terms on the right side of equation (1) denote the transport of DOC by the combination of hydrodynamic dispersion (due to soil heterogeneity) and molecular diffusion (due to the concentration gradient) and by advection (due to liquid water movement), and these processes together define the transport of DOC within soil columns and the export of DOC from soil columns. Please refer to Fan et al. [27] for the detailed derivation of equation (1).

The concentration of PDOC (i.e., *S_PDOC_*) is defined as:
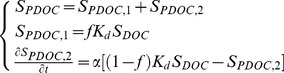
(2)where *S_PDOC,1_* represents the PDOC associated with type 1 exchange sites (instantaneous sorption/desorption), *S_PDOC,2_* represents the PDOC associated with type 2 exchange sites (kinetic sorption/desorption), *f* is the fraction of exchange sites in equilibrium with type 1 sites ranging from 0 to 1 (unitless), *K_d_* is a linear partition coefficient between the solid and aqueous phase (mL g^−1^), and *α* is the mass transfer rate (h^−1^). If the product of (1– *f*)*K_d_S_DOC_* is greater than *S_PDOC,2_*, the mass transfer rate (i.e., *α*) represents the sorption rate of DOC; otherwise, the mass transfer rate represents the desorption rate of DOC. The two-site chemical nonequilibrium model can be reduced to a one-site model with either an exclusively nonequilibrium process by setting *f* = 0 or an exclusively equilibrium process by setting *f* = 1.

The pore water velocity, *v*, was calculated as:
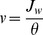
(3)where *J_w_* is the unsaturated water flux density (cm h^−1^) defined as:
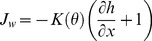
(4)where K(*θ*) is the unsaturated hydraulic conductivity (cm h−1) and *h* is the soil matric potential (cm). The unsaturated hydraulic conductivity, K(*θ*), is a function of volumetric moisture content (*θ*) and calculated using the Mualem-van Genuchten model defined as [32], [33]:



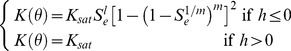
(5)

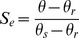
(6)

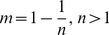
(7)where *K_sat_* is the saturated hydraulic conductivity (cm h^−1^), *S_e_* is the soil effective saturation (cm^3^ cm^−3^), *l* is an empirical pore-connectivity parameter, *m* and *n* are empirical pore-size distribution parameters, *θ_r_* is the residual moisture content (cm^3^ cm^−3^), and *θ_s_* is the saturated moisture content (cm^3^ cm^−3^). The parameter, *l*, is fixed to 0.5 following Mualem [32]. The Mualem-van Genuchten model parameters, *K_sat_*, *m*, *θ_r_*, and *θ_s_*, were set to 1017.4, 2.38, 0.04, and 0.95 for live moss [34]; 100.8, 0.01, 0.93, and 1.9 for dead moss and lightly decomposed SOC; 0.716, 0.18, 0.88, and 1.7 for moderately decomposed SOC; and 0.036, 0.22, 0.83, and 1.6 for well-decomposed SOC [35].

The soil temperature and moisture dependencies of decomposition for DOC and SOC, *F*(*T*, *M*), were derived from laboratory incubation experiments conducted under various soil temperature and moisture conditions [36]. *F*(*T*, *M*) is defined mathematically with the following equation [25]:
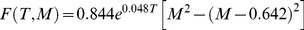
(8)


The upper boundary condition of equation (1) is defined as a third-type boundary condition [37]:
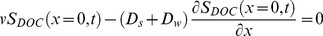
(9)


The Neumann boundary condition is used as the lower boundary condition of equation (2) and defined as [37]:
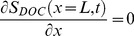
(10)where *L* is the thickness of the SOC horizons (cm).

### Soil Organic Carbon Pool

The SOC pool depends on SOC loss due to hydrolysis and SOC gain due to both microbial necromass and external SOC input and is quantitatively expressed as:
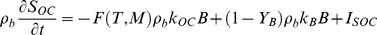
(11)where *S_OC_* is the mass of SOC in a given soil layer (g cm^−2^) and *I_SOC_* is the external SOC input (e.g., litterfall and root turnover; g h^−1^).

### Microbial Biomass Carbon Pool

The MBC pool depends on MBC loss due to both microbial death and maintenance respiration and MBC gain due to microbial growth and is quantitatively expressed as [28], [38]:
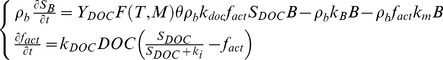
(12)where *Y_DOC_* is the fraction of DOC that transfers to microbial biomass during turnover (unitless), *k_m_* is the microbial maintenance respiration rate (h^−1^), and *k_i_* is the microbial inhabitation constant (unitless). The above equation assumes that the microbial maintenance respiration is not as sensitive to soil temperature and moisture as microbial growth [39]. This assumption is not unreasonable since microbial maintenance respiration was detected in tundra soils at a soil temperature of −39°C when there was no microbial growth [40].

### CO_2_ flux

The total heterotrophic CO_2_ produced in the soil-water systems depends on SOC hydrolysis, microbial growth, and microbial maintenance respiration and is defined as:
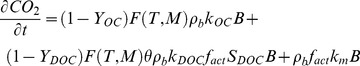
(13)


The first, second, and third terms on the right side of above equation represent the CO_2_ produced through hydrolysis, DOC uptake by microbes (i.e., microbial growth), and microbial maintenance respiration, respectively.

### Model Parameterization

The density of SOC in each soil layer was initialized based on the simulation results of inverse modeling with a multi-pool SOC model (composed of fine, coarse, and humic SOC pools), which simulates SOC dynamics for an ecosystem undergoing growth-burning-growth cycles since the retreat of the Laurentide ice sheet to the present [25], [41]. During the model inversion, differences between the simulated and measured total SOC mass, SOC thickness, and carbon isotope (^14^C) profiles were minimized by estimating SOC decomposition rates with a global optimization strategy (i.e., stochastic ranking evolutionary strategy [25], [41]). After the decay rates for the SOC pools were estimated, the density of SOC in each soil layer was calculated using those estimated rates.

The concentration profiles of DOC in each soil layer were initialized on the basis of field-observed DOC concentration profiles [27]. Information on total DOC input for both sites is very limited. Thus, three different annual DOC inputs were used to drive the model, and these inputs were set equal to 10%, 30%, and 50% of the annual net primary production (NPP) [42]. The annual NPP was approximately 268 and 250 g OC m^−2^ yr^−1^ for WD and MWDp, respectively [25]. Other important factors controlling the DOC input to soil (such as, when/how often [e.g., frequency] and how much [e.g., intensity]) are also unknown in boreal ecosystems. Thus, we assumed that an input of DOC to the soil occurred every 3, 6, 12, 18, 30, 36, 40, 60, 90, 120, 180, 225, 300, 360, 450, 600, 720, 900, 1200, or 1800 hours to evaluate a range of possible frequencies in a sensitivity analysis. The corresponding intensity of DOC input was calculated by dividing the total DOC input by the frequency. The SOC input, *I_SOC_* in equation 11, was set equal to the observed belowground NPP, i.e., 134 g OC m^−2^ yr^−1^ for WD and 116 g OC m^−2^ yr^−1^ for MWDp [25], [43] because litterfall and root turnover data were unavailable for these sites.

The microbial biomass was initialized to represent 3% of SOC at a given soil depth [38]. However, little information is available on the quality of microbial necromass not only for boreal ecosystems but also for temperate ecosystems. In the model, the quality of microbial necromass is associated with the fraction of microbial necromass that transfers to the DOC pool (parameter *Y_B_* in equation 1). To cover most of the possible values of this parameter (i.e., *Y_B_*), eleven different values (0.0, 0.1, 0.2, 0.3, 0.4, 0.5, 0.6, 0.7, 0.8, 0.9, and 1.0) are assigned to *Y_B_*.

The parameters associated with the transport and sorption/desorption of DOC were assigned based on the results of Fan et al. (2010). The parameters associated with the fate of SOC, DOC, and MBC were assigned based on the results of Blagodatsky et al. (2010) and Allison et al. (2010).

The model (computer code is available from the authors upon request) was run at an hourly time step for the growing season (May 1^st^ to September 30^th^). The model with the initialized OC pools was first run to reach equilibrium (equilibrium run) with the default parameters (Table 1). Annual average soil temperature and moisture profiles were used to drive the model during the equilibrium runs. After the soil pools (i.e., SOC, DOC, and MBC) reached equilibrium, the model was run with the hourly measured soil temperature and moisture to simulate the priming effects with three different DOC inputs: 10%, 30%, and 50% of the annual NPP and various DOC input frequencies and intensities as identified earlier. In addition, an increase by 3°C in soil temperature throughout the profile was added to the DOC simulations to explore how future warming might alter the role of priming in boreal forests.

**Table 1:** Default parameter values used in model simulations.

Parameter	Unit	Value	Equation number where parameter first appears
*α*	h^−1^	0.274^1^	2
*f*	–	0.317^1^	2
*K_d_*	L g^−1^	0.136^1^	2
*k_OC_*	day^−1^	0.019^2^	1
*Y_OC_*	–	0.02^2^	1
*k_DOC_*	day^−1^	6590^2^	1
*Y_DOC_*	–	0.62^2^	12
*k_B_*	h^−1^	0.0002^3^	1
*k_m_*	day^−1^	0.01	12
*k_i_*	–	0.00264^2^	12
*Y_B_*	–	0.5^2^	1

1 Fan et al. [27];

2 Blagodatsky et al. [28];

3 Allison et al. [38].

See text for more complete information and definitions of parameters; *α* is the mass transfer coefficient; *f* is the fraction of exchange sites in equilibrium with type 1 sites; *K_d_* is the linear partition coefficient between SOC and DOC; *k_OC_* is the hydrolysis rate coefficient of SOC; *Y_OC_* is the fraction of hydrolyzed SOC that transfers to the DOC pool during turnover; *k_DOC_* is the decomposition rate of DOC; *Y_DOC_* is the fraction of DOC that transfers to microbial biomass during turnover; *k_B_* is the microbial death rate; *k_m_* is the microbial maintenance respiration rate; *k_i_* is the microbial inhabitation constant; *Y_B_* is the fraction of microbial biomass that transfers to the DOC pool during turnover.

### Sensitivity Analysis

A sensitivity analysis was conducted by varying parameters over one order of magnitude while holding the remaining parameters constant. The range of values used in the sensitivity analysis should be large enough to cover most of possible parameter values and yet have a physical meaning. The sensitivity index (*SI*) is expressed as [44]:
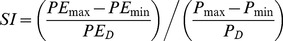
(14)where *PE_max_*, *PE_min_*, and *PE_D_* are the simulated maximum, minimum, and reference priming effects, respectively; *P_max_*, *P_min_*, and *P_D_* are the maximum, minimum, and default parameter values, respectively.

## Results

The simulated heterotrophic respiration induced by DOC inputs equal to 10% and 30% of annual NPP fell within the ranges of heterotrophic respiration measured at these sites (67–151 g C m^−2^ for MWDp and 91–189 g C m^−2^ for WD) under the assumption that heterotrophic respiration contributed to 40–60% of total soil respiration [26], [45], while the simulated heterotrophic respiration induced by DOC input equal to 50% of annual NPP was greater than the upper limit of measured heterotrophic respiration. Therefore, only simulations with DOC input equal to 10% and 30% of annual NPP were included in the reported results, but the simulations with DOC input equal to 50% of annual NPP were included in the figures for the purpose of comparison.

**Figure 3:**
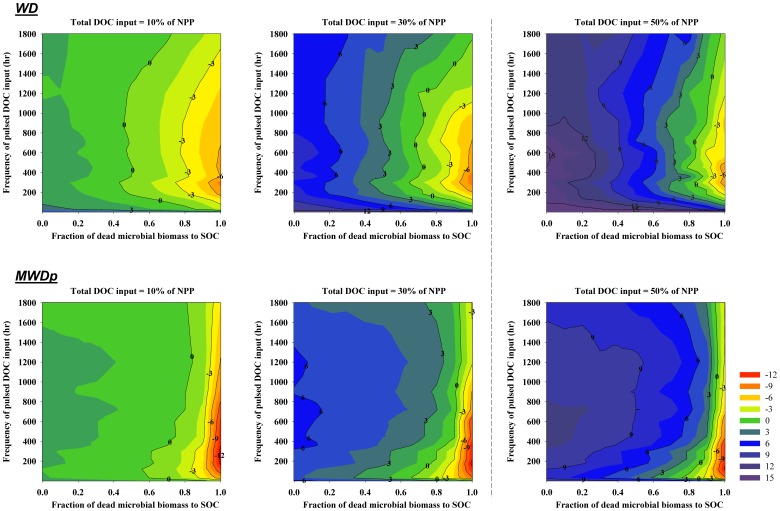
Variations in simulated priming effects (g OC m^−2^ yr^−1^) associated with different amounts of total DOC input at each site (WD and MWDp) as affected by different frequencies and intensities of DOC input (Y axis) and the quality of microbial necromass (as represented by the fraction of microbial necromass apportioned to SOC). The simulated heterotrophic respiration induced by DOC input equal to 50% of annual NPP was greater than the upper limit of measured heterotrophic respiration, but was included for comparison purposes.

Our model results indicated that the amount of SOC released to the atmosphere as CO_2_ due to priming ranged from −8.6 (negative priming) to 14.8 g OC m^−2^ yr^−1^ (positive priming) for WD and from −12.7 to 6.4 g OC m^−2^ yr^−1^ for MWDp (Figure 3). The maximum percentages contributed by positive priming to the total CO_2_ efflux were 20% for WD and 12% for MWDp. The maximum percentages contributed by negative priming effects to total CO_2_ efflux were 12% for WD and 10% for MWDp. In the model, priming effects strongly depended on (1) the frequency and intensity of external DOC input to the soil and (2) the fraction of microbial necromass allocated to the SOC pool. For a given frequency of pulsed external DOC input, both sites tended to have negative priming of SOC when most of microbial necromass was transferred to SOC. When most of microbial necromass was transferred to DOC, the external DOC input tended to have strong positive priming effects on SOC decomposition. However, the frequency of pulsed DOC input also affected priming. For a given proportion of microbial necromass allocated to DOC, an optimal frequency of pulsed DOC input maximized positive or negative priming effects on SOC decomposition. Frequencies of DOC input above or below the optimal values weakened the positive or negative priming.

**Figure 4:**
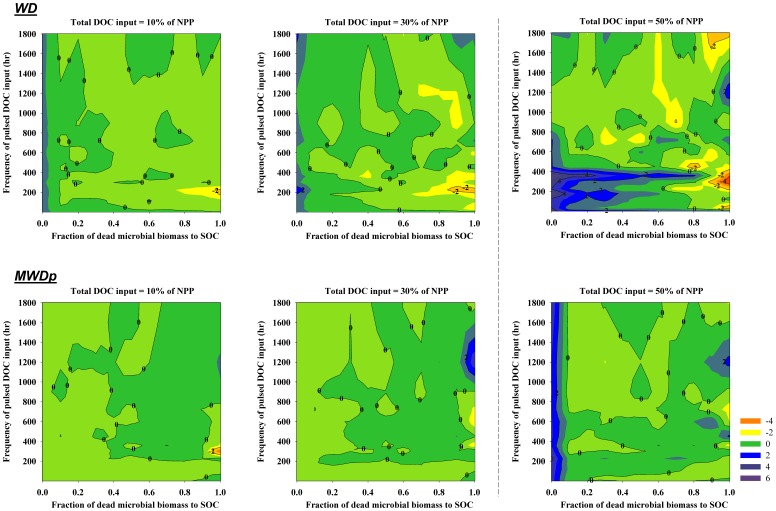
Variations in simulated impacts of water transport on priming effects (g OC m^−2^ yr^−1^) associated with different amounts of total DOC input at each site (WD and MWDp) as affected by different frequencies and intensities of DOC input (Y axis) and the quality of microbial necromass (as represented by the fraction of microbial necromass apportioned to SOC). The simulated impacts are indicated as the differences between the simulations with water transport and simulations without water transport.

DOC can be transported by advection due to water movement and by diffusion due to a concentration gradient and the combination of these two processes determines the distribution of DOC in the soil profile. To investigate how important the movement or distribution of DOC is to priming, the pore water velocity and diffusion coefficient were manually set equal to zero in another set of model simulations. The results (Figure 4) indicated that the movement of DOC had relatively strong impacts on both negative and positive priming for the well-drained WD site without permafrost (−2.6 to 3.0 g OC m^−2^ yr^−1^) but had smaller impacts on priming for the moderately well-drained MWDp site with permafrost (−1.7 to 1.0 g OC m^−2^ yr^−1^). For both sites, DOC transport had a greater impact on priming if DOC input was equal to 30% of NPP than if DOC input equaled 10% of NPP.

**Figure 5:**
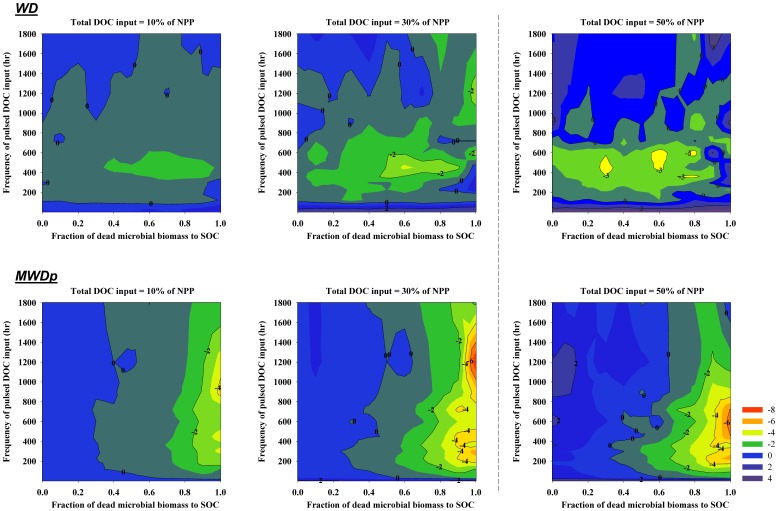
The simulated impacts of 3°C warming on priming effects (g OC m^−2^ yr^−1^) associated with different amounts of total DOC input at each site (WD and MWDp) as affected by different frequencies and intensities of DOC input (Y axis) and the quality of microbial necromass (as represented by the fraction of microbial necromass apportioned to SOC). The simulated impacts are indicated as the differences between the simulations with warming and simulations without warming.

The model results showed strong relationships between priming effects and soil warming, with greater impact of warming at the site with permafrost (MWDp) compared to the WD site with no underlying permafrost (Figure 5). There were clear patterns for the MWDp site, where the magnitude of both positive and negative priming became greater when soil temperature increased. However, such patterns were different for the WD site, where soil warming tended to decrease priming effects when the frequency of pulsed DOC input was moderate and increase priming effects for other DOC input frequencies. At both sites, soil warming impacts on priming effects were greater when DOC inputs were equal to 30% of NPP compared to effects when DOC inputs were only 10% of NPP.

Sensitivity analyses (Table 2) showed that variation in the hydrolysis rate coefficient of SOC (*k_OC_*) had the greatest effect on priming for both sites, followed by variations in microbial death rate (*k_B_*) and microbial maintenance respiration rate (*k_m_*). Variations in parameters associated with the sorption/desorption of DOC including the mass transfer coefficient (*α*), fraction of exchange sites in equilibrium with type 1 sites (*f*), and the linear partition coefficient between SOC and DOC (*K_d_*) had relatively small impacts on priming.

**Table 2:** Sensitivity indices for model parameters^1^.

Parameter	Sensitivity index (unitless)	
	WD	MWDp
*α*	0.002	0.046
*f*	0.004	0.001
*K_d_*	0.010	0.152
*k_OC_*	12.850	28.000
*Y_OC_*	0.183	0.497
*k_DOC_*	0.254	0.798
*Y_DOC_*	0.536	1.045
*k_B_*	1.097	2.641
*k_m_*	1.038	2.739
*k_i_*	0.318	1.016

1 Please refer to the main text and Table 1 for the definitions of parameters. The sensitivity index was calculated with equation (14).

## Discussion

Our approach to modeling priming effects in boreal forest regions is unique because it combines a number of features that, to our best knowledge, have not been included together in a single process-based model before. In our model, priming effects are explicitly tied to soil DOC and microbial biomass dynamics that are strongly related to soil hydrology (e.g., unsaturated water flow) and soil physical properties (e.g., sorption/desorption, soil water retention curve). DOC is vitally important to microbial dynamics; therefore representations of the coupled unsaturated water flow and DOC movement or re-distribution within the soil profile are important for properly simulating soil microbial biomass dynamics and thus subsequent priming effects under field conditions.

### Biological Factors Controlling Priming Effects

The most important finding that emerged from our modeling exercises was that priming effects were most sensitive to variations in the hydrolysis process, the first and most important step in breaking down larger SOC molecular compounds (e.g., lipids, lignin, polysaccharides) into smaller molecular compounds (e.g., sugars, amino acids, fatty acids) [46]. This finding suggests that variations in the inherent quality or composition of SOC might be the most important factor controlling variations in priming effects for boreal forest soils. Hence, priming effects are likely to be strongly variable across different boreal ecosystems (e.g., black spruce vs. peatland). To the best of our knowledge, no studies have investigated how priming effects vary for boreal ecosystems with different SOC quality. Our results also suggest that variations in the spatial distribution of SOC quality might be used to upscale priming effects from laboratory or site scales to larger spatial scales across boreal regions.

Even within the same or similar ecosystems, our sensitivity analyses suggested that variations on soil microbial community composition and structure might have a strong impact on priming effects through variations on the quality of microbial necromass (i.e., the relative apportionment of microbial necromass between DOC and SOC pools). Although incorporation of microbial necromass into the SOC pool has been recently emphasized as an important channel for SOC stabilization and sequestration [47], [48], [49], [50], very limited knowledge is available regarding how and to what extent microbes access different OC pools in soils, such as the DOC, PDOC and SOC pools, or how their necromass is distributed amongst these pools. On the other hand, changes in microbial biomass dynamics also alter microbial maintenance respiration and decomposition rates of SOC and DOC pools [28]. Further experimental and modeling studies are needed to link the decomposition rates of different pools with the fate of microbial necromass as well as variations in the dynamics of microbial communities (quantitatively and qualitatively) across time and space in relation to variations in soil moisture dynamics.

Our model results also suggest that the frequency and intensity of external DOC input is an important modulator of the magnitude and direction of the priming effects. Hamer and Marschner [4] reported that multiple small additions of DOC caused more positive priming effects than a single large addition. However, the quality of DOC input (e.g., DOC originating from litter vs. root exudates) under field conditions is rather complicated and strongly related to many ecosystem variables and states, including vegetation type and growth stage, rainfall intensity and duration, topography (e.g., slope), soil hydrological condition, etc. Our model sensitivity analyses indicated that variations in DOC decomposition rate (*k_DOC_*) had moderate impacts on priming effects, suggesting that variations on the quality of DOC input may affect priming to some extent but not as strongly as variations in inherent SOC quality and microbial biomass dynamics (i.e., microbial death rate and maintenance respiration).

### Physical and Hydrological Factors Controlling Priming Effects

**Figure 6:**
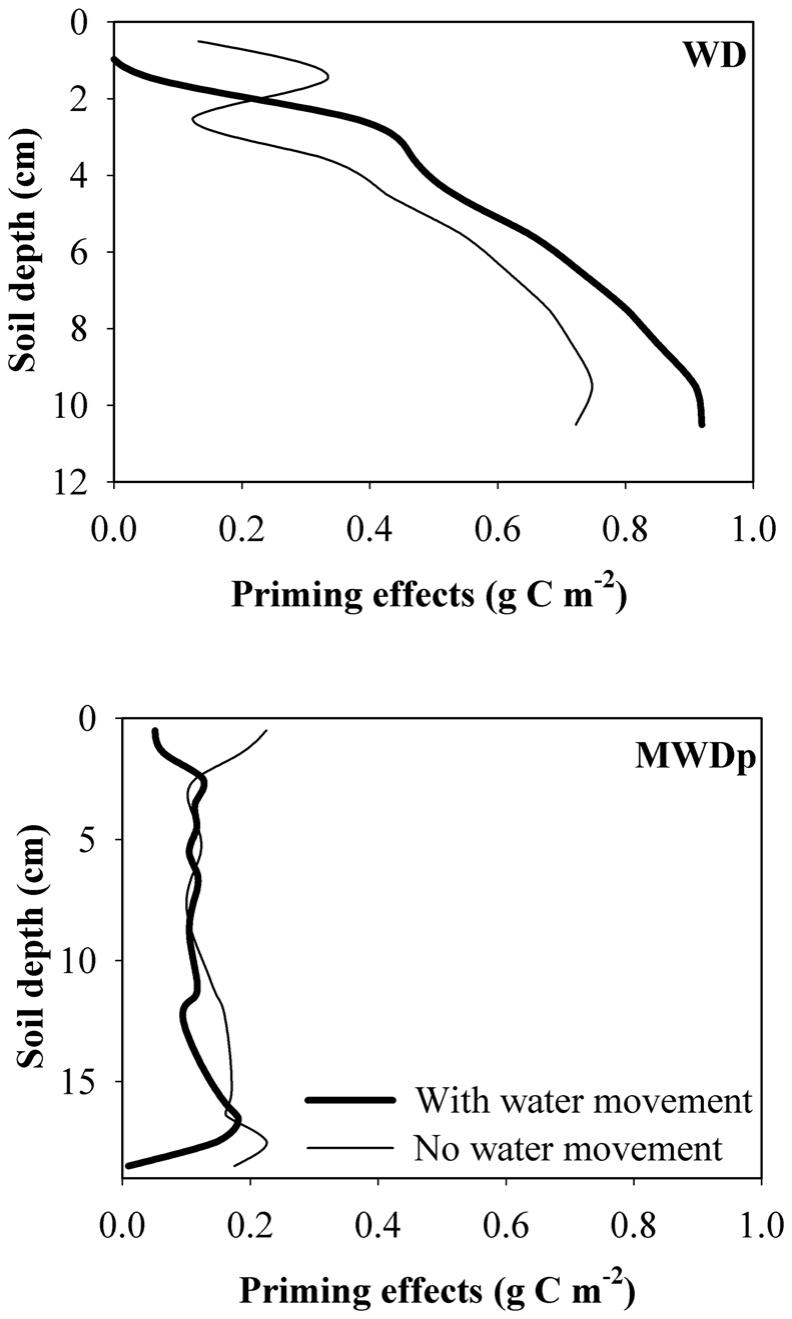
Simulated priming effects throughout the soil profile at each site (WD and MWDp) with total DOC input equal to 30% of NPP, DOC input occurring every 60 hours, and the fraction of microbial necromass apportioned to SOC equal to 0.5. The curves “with water movement” were derived based on the equation (1) while the curves “no water movement” were derived by setting the pore water velocity and diffusion coefficient equal to zero in equation (1).

The WD and MWDp sites differ in their precipitation patterns, drainage, and subsequent unsaturated water movement and soil moisture content (Figure 1). At the WD site, transport of DOC played an important role in priming effects due to favorable conditions for water movement. These conditions cause a large amount of DOC to be transported to deeper soil layers where large amounts of SOC are stored due to the higher bulk densities of deeper layers. Thus, greater DOC transport enables increased priming of SOC in those deep soil layers (Figure 6). However, enhanced transport of DOC from surface to deeper soil layers also decreased the priming effects produced by DOC in surface soil (Figure 6). Therefore, the overall impacts of DOC transport on total priming effects within the profile at the WD site depend on the balance between decreases in priming effects at the surface and increases for deeper soil layers. In comparison, DOC transport played a less important role in priming at the MWDp site due to unfavorable conditions for water movement. Under these conditions, slow diffusion-induced transport of DOC dominates the movement and distribution of DOC. Consequently, most of the DOC input to a given SOC layer from both external and internal sources tends to stay in the same layer. As a result, DOC transport had little impact on priming effects at the MWDp site (Figure 6).

Taken together, our results demonstrate how water movement can impact priming effects under field conditions by directly redistributing DOC through the soil profile. Indeed, water movement was substantially responsible for the larger total profile priming effects at the WD site. Furthermore, water movement can indirectly affect priming effects via impacts on soil thermal and water dynamics [51], which may be partially responsible for the differences in observed soil temperature and moisture between the WD and MWDp sites. At the WD site (with no underlying permafrost), the temperature and moisture content of deep soil layers are more favorable for microbial activity and thus priming effects (Figure 1). This may partially explain why priming effects increase with soil depth (even without DOC transport) at this site (Figure 6). In contrast, at the MWDp site, priming effects were relatively invariable with soil depth (Figure 6), and it appears that this is related, at least in part, to smaller differences in soil temperature and moisture between surface and deep soil depths compared to the WD site.

In our model, the sorption and desorption processes were assumed to be reversible. When the concentration of DOC is below the partitioning concentration between PDOC and DOC, PDOC currently in the solid phase is released from the SOC to the aqueous phase to become DOC (desorption). When the concentration of DOC is above the partitioning concentration, the DOC returns to the PDOC phase (sorption). These dynamics of the sorption/desorption processes maintained relatively stable DOC concentrations within the model. As a result, the parameters associated with sorption/desorption had limited impacts on priming effects. The assumption that sorption/desorption processes are reversible is reasonable for our modeling exercises due to the limited amount of mineral particles in the OC horizons. However, an irreversible (or only slowly reversible) sorption/desorption mechanism might have greater impacts on DOC dynamics, and thus priming effects, in the underlying mineral horizon due to the interactions between SOC and mineral particles. For example, DOC might be physically protected in the fine-scale pores of microaggregates or chemically bound to mineral particles (e.g., via cation bridging and/or ionic attraction) [52], [53], [54]. Moreover, the interactions between DOC and fire-derived SOC (i.e., black carbon) might be potentially important to the dynamics of DOC due to the strong sorption of DOC to black carbon, which was not considered in our model but might have great impacts on priming effects [55].

### Response of Priming Effects to Climate Change

Projected increases in atmosphere CO_2_ concentration will likely increase NPP and thus the production of DOC [56]. The simulations with DOC input equal to 50% of NPP (Figure 3) indicated that an increase in DOC input will significantly increase both negative and positive priming effects. Our simulation results with soil warming (Figure 5) indicated that warming tends to increase the magnitude of priming effects. Taken together, these simulations suggest that priming effects will likely become stronger with the warming climate. However, climate change may significantly alter vegetation dynamics (structure and diversity) by changing the soil hydrological, physical, and chemical conditions (e.g., permafrost degradation, soil nutrient condition, etc.), which may significantly change soil microbial community composition and dynamics [57], the quality of litterfall OC, and the subsequent quality of SOC [58]. All of these changes could significantly impact DOC inputs.

Poorly drained sites (e.g., peatlands), which are usually underlain with permafrost, were not investigated in this study due to the limited data available for such sites. In the context of climate change, dry or wet thermokarst may form in poorly drained sites after the permafrost thaws depending on soil drainage conditions [59], which may change the surface topography and thus water, nutrient, and heat transport. Agafonov et al. [60] suggested that warming probably triggered the permafrost thaw during the 1920s and 1930s in Western Siberia, Russia. However, thermokarst development since the 1950s in this region was strongly related to a change in precipitation rather than to warming, suggesting that the amount and distribution of precipitation may be more important than warming to peatland SOC dynamics. Similarly, our modeling results suggest that greater water movement through the soil profile may carry DOC to deeper SOC layers and effectively release more CO_2_ due to enhanced priming effects. Consequently, we might predict that CO_2_ released by priming effects will increase for boreal sites with dry thermokarst formation, due to the creation of more favorable conditions for water flow. In contrast, the CO_2_ released by priming effects will likely decrease for sites where wet thermokarst forms due to the limited water flow.

Another unique factor shaping the boreal ecosystem is fire. Most of SOC in organic horizons will be lost to the atmosphere as CO_2_ during fire events. The removal of organic layers will substantially change the soil thermal (e.g., permafrost degradation) and hydrological dynamics and thus will affect post-fire vegetation re-growth [61], [62]. Vegetation re-growth will, in turn, affect SOC settings (e.g., thickness and mass) and the subsequent soil thermal and hydrological conditions (e.g., permafrost re-growth). Therefore, post-fire vegetation dynamics, including the replacement of small plants/shrubs with large ones, is a result of complex interactions between vegetation dynamics and soil processes [63]. How priming effects will vary during vegetation succession and how climate change will affect such variation are still poorly understood. These are certainly research areas where more field experimental data and modeling are needed.

## Conclusions

Priming effects have been mostly studied in the laboratory with soils from agricultural or natural ecosystems (e.g., forest) located in temperate environments. Information on priming effects is extremely limited for boreal ecosystems. Our coupled process-based DOC and microbial biomass dynamics model, to the best of our knowledge, is the only model to simultaneously simulate the various fate and transport processes of DOC and microbial biomass dynamics under field conditions. Our model simulations showed that priming effects were more sensitive to inherent SOC quality and the microbial community than to physical properties (e.g., sorption/desorption) in boreal forest soils, in large part because most SOC is found in organic horizons with low mineral contents. Our results also suggested that the redistribution of DOC within soil profiles due to water transport was also a key factor determining the overall priming effects.

Considering that the SOC accumulation rate in boreal black spruce forests ranges from 20 to 40 g SOC m^−2^ yr^−1^ for stand ages of less than 200 years [64], a few grams to a few dozen grams of SOC loss per year due to priming effects might significantly affect ecosystem carbon, water, and energy balances. However, because the parameters used to simulate the decomposition of DOC and SOC in our model were obtained from laboratory studies of soils from temperate ecosystems, caution is warranted. Decomposition rates for boreal ecosystems may be different from those obtained in temperate ecosystems. Our sensitivity analyses indicated that small changes in some of these parameters may have great impacts on the model output. Therefore, the actual priming effects may be different from our model simulations. Also, several factors and mechanisms that might significantly impact priming effects were not included in our model – such as soil chemistry (e.g., pH), soil nutrient (e.g., nitrogen) availability, competition among microorganisms or between microorganisms and living roots for nutrients, and the influences of soil drying-wetting and freezing-thawing cycles on priming effects [1], [16]. Critical evaluation of these factors and mechanisms needs to be addressed in future model development. Nonetheless, our fully coupled DOC and microbial biomass dynamics model can be used as a useful prototype tool to more accurately simulate future SOC dynamics in boreal ecosystems under a changing climate as data needed to validate the model (e.g., field-based priming data) become available in the future.
